# Air pollution, methane super-emitters, and oil and gas wells in Northern California: the relationship with migraine headache prevalence and exacerbation

**DOI:** 10.1186/s12940-021-00727-w

**Published:** 2021-04-17

**Authors:** Holly Elser, Rachel Morello-Frosch, Alice Jacobson, Alice Pressman, Marianthi-Anna Kioumourtzoglou, Richard Reimer, Joan A. Casey

**Affiliations:** 1 Stanford University School of Medicine, Stanford Center for Population Health Sciences, Stanford, USA; 2 Department of Environmental Science, Policy, and Management and School of Public Health, University of California Berkeley, Berkeley, CA USA; 3 Research, Development and Dissemination, Sutter Health, Sacramento, USA; 4 Department of Environmental Health Sciences, Mailman School of Public Health, Columbia University, 722 W 168th St, Rm 1206, New York, NY 10032-3727 USA; 5 Department of Neurology and Neurological Science, Stanford University School of Medicine, Stanford, USA

**Keywords:** Electronic health records, Migraine, Methane, oil and gas fields, Nitrogen dioxide, Particulate matter, Environmental exposure

## Abstract

**Background:**

Migraine–an episodic disorder characterized by severe headache that can lead to disability–affects over 1 billion people worldwide. Prior studies have found that short-term exposure to fine particulate matter (PM_2.5_), nitrogen dioxide (NO_2_), and ozone increases risk of migraine-related emergency department (ED) visits. Our objective was to characterize the association between long-term exposure to sources of harmful emissions and common air pollutants with both migraine headache and, among patients with migraine, headache severity.

**Methods:**

From the Sutter Health electronic health record database, we identified 89,575 prevalent migraine cases between 2014 and 2018 using a migraine probability algorithm (MPA) score and 270,564 frequency-matched controls. Sutter Health delivers care to 3.5 million patients annually in Northern California. Exposures included 2015 annual average block group-level PM_2.5_ and NO_2_ concentrations, inverse-distance weighted (IDW) methane emissions from 60 super-emitters located within 10 km of participant residence between 2016 and 2018, and IDW active oil and gas wells in 2015 within 10 km of each participant. We used logistic and negative binomial mixed models to evaluate the association between environmental exposures and (1) migraine case status; and (2) migraine severity (i.e., MPA score > 100, triptan prescriptions, neurology visits, urgent care migraine visits, and ED migraine visits per person-year). Models controlled for age, sex, race/ethnicity, Medicaid use, primary care visits, and block group-level population density and poverty.

**Results:**

In adjusted analyses, for each 5 ppb increase in NO_2_, we observed 2% increased odds of migraine case status (95% CI: 1.00, 1.05) and for each 100,000 kg/hour increase in IDW methane emissions, the odds of case status also increased (OR = 1.04, 95% CI: 1.00, 1.08). We found no association between PM_2.5_ or oil and gas wells and migraine case status. PM_2.5_ was linearly associated with neurology visits, migraine-specific urgent care visits, and MPA score > 100, but not triptans or ED visits. NO_2_ was associated with migraine-specific urgent care and ED visits, but not other severity measures. We observed limited or null associations between continuous measures of methane emissions and proximity to oil and gas wells and migraine severity.

**Conclusions:**

Our findings illustrate the potential role of long-term exposure to multiple ambient air pollutants for prevalent migraine and migraine severity.

**Supplementary Information:**

The online version contains supplementary material available at 10.1186/s12940-021-00727-w.

## Introduction

Migraine is an episodic disorder characterized by severe headache often associated with nausea or sensitivity to light and sound. In 2016, the estimated global prevalence of migraine was 14.4% with over 1.04 billion individuals affected [1]. In the United States (U.S.), migraine is most common among individuals aged 30 to 39 and follows a social gradient wherein migraine is less common among wealthier individuals [2, 3]. Migraine can lead to disability; in the U.S., estimated annual costs associated with migraine range from $13 to 16.6 billion annually due to lost productivity, work and school absences, and short-term disability [4–7].

Given the episodic nature of migraine headache, considerable attention has been paid to the study and identification of common triggers. Among the most frequently self-reported triggers of migraine are sleep disturbances and fatigue; stress or relief of stress; menstruation and pregnancy; smoking; and food and alcohol [8–12]. Factors such as noise, season, and weather variations have also been implicated as migraine triggers [8, 13–15]. Examples of common sources of environmental noise that may precipitate a migraine attack include traffic-related noise from roads, railways, aircrafts, and parking cars [16]. Individuals with migraine frequently attribute their headaches to weather variations, including changes in temperature and barometric pressure [11, 13, 17–19].

Research to date also implicates short-term exposure to a variety of air pollutants as triggers for migraine headache. Fine particulate matter (PM_2.5_) is among the most frequently studied pollutants; increased levels of PM_2.5_ have been associated with more frequent migraine-specific emergency department (ED) visits in Canada, Taipei, and South Korea [20–24], although, a case-crossover study of 7054 patients in Boston reported no significant association with ED visits [25]. In a time-series study of 1059 ED visits recorded at a Vancouver hospital, levels of sulfur dioxide (SO_2_) were associated with ED visits for migraine [26]. Levels of ozone, carbon monoxide, nitrogen dioxide (NO_2_), and coarse particles (PM_10_) have also been linked with migraine-specific ED visits in case-crossover studies based on daily clinic data from 1000,000 patients from the National Health Insurance Program in Taiwan [23, 24]. A cross-sectional survey of 7785 primary care patients of the Geisinger Clinic in 2014 found that individuals exposed to the highest levels of unconventional natural gas development were more likely to have migraine headache [27]. Unconventional natural gas development can produce PM_2.5_, volatile organic compounds (VOCs), noise and light pollution, and stressful community changes that could trigger migraine [28].

To date, few studies have considered the implications of long-term exposure to common environmental pollutants–which may capture potential residential disparities in the burden of headache based on local average air quality–and no analyses have been conducted in the Western U.S. or on specific air pollution sources. Recently, the California Air Resources Board (CARB) conducted an air survey of methane super-emitters, point sources of methane emissions, including dairies, landfills, refineries, and oil and gas infrastructure [29]. These facilities emit a variety of co-pollutants such as SO_2_, hydrogen sulfide, PM_2.5_, and VOCs [30–32], and the new CARB data provide an opportunity to assess their implications for migraine. California is also a top-10 U.S. producer of crude oil, with over 200,000 oil and gas wells drilled in the state [33].

The present study leverages data from the Sutter Health electronic health record (EHR) database in Northern California and builds on prior research linking air pollutants and migraine headache. Our analyses include an expanded set of exposure measures, including long-term ambient PM_2.5_ and NO_2_ concentrations, methane emissions, and active oil and gas wells as measured at the beginning of the study period. We selected these exposures, particularly methane super-emitters and active oil and gas wells, because if linked to migraine, policies could reduce emissions at the source. Whereas past research has largely relied on migraine-specific ED visits as a crude proxy for severe headache, we incorporate additional measures of headache severity. We conducted a case-control study to ascertain whether migraine case status was associated with long-term exposure to any of the four environmental exposure measures as compared with controls. Next, we conducted a case-case analysis to ascertain whether environmental exposures were associated with more severe headache among individuals with established diagnosis of migraine. We hypothesized a priori that environmental exposures would be associated with both migraine case status and with disease severity.

## Methods

We conducted a case-control study and case-case analysis to examine the relationship between migraine severity and exposures of interest. This approach was selected based on computational feasibility, and because disease-based sampling is efficient when multiple exposures are considered and when the outcome of interest is relatively rare [34]. Cases and controls were identified through the Sutter Health EHR database. Sutter Health is a large, mixed-payer, integrated healthcare system in Northern California that delivers comprehensive medical services through its network of 24 acute-care hospitals and more than 100 ambulatory clinics. Approximately 3.5 million patients receive care through Sutter each year at hospitals and clinics located in 22 counties; our study subjects resided in 27 urban and rural counties. Sutter’s Epic EHR (Epic Systems Corporation, Verona, Wisconsin) is fully integrated across all hospital and ambulatory sites. Data for cases and controls were retrospectively extracted from the Sutter EHR for the study period between January 1, 2014 and December 31, 2018.

Patient demographic data from the EHR included sex (male, female), race/ethnicity (non-Hispanic Asian, Black, white, other, or Hispanic); and marital status (divorced, separated, widowed; married or partnered; single; other or unknown). We used date of birth to compute age in years at the start of follow-up. Health characteristics extracted from the EHR included whether the individual was a Medicaid beneficiary (yes, no); body mass index (BMI) category in kg/m^2^ [less than 18.5 (underweight); 18.5–24.9 (normal); 25–29.9 (overweight); 30–34.9 (obese class 1); 35–39.9 (obese class 2); 40 or more (obese class 3)]; number of and reason for primary care, specialty care, urgent care, and emergency department visits. We assigned residential address for the study period (2014–2018) based on address of record in October 2019. Using assigned residential address, we linked block group-level percent living below the federal poverty threshold and population density (individuals per km^2^) using data from the 2014–2018 American Community Survey.

### Migraine case ascertainment and control selection

Both cases and controls were selected from the study base of eligible patients over the age of 18 with at least one primary care encounter during the five-year study period (2014–2018) that resided in one of 27 counties in Northern California. We ascertained case status using the Migraine Probability Algorithm (MPA), a validated approach for identification of individuals diagnosed with migraine from EHR data [35]. Briefly, a numeric score that ranges from zero to 101 is calculated based on the following criteria: encounters (hospital inpatient, emergency room and outpatient) with a primary or secondary diagnostic code for migraine from the *International Classification of Diseases*, Ninth Revision (*ICD*-9 346.xx) or Tenth Revision (*ICD*-10 G43.xxx); an *ICD*-9 or *ICD-10* code for migraine in the patient’s Significant Health Problem List (SHP); and filled prescriptions for migraine-specific abortive medications (i.e., triptans, ergotamines). An MPA score greater than 10 is consistent with diagnosis of migraine. We selected three controls for every case from the Sutter EHR database. Controls were frequency matched to cases based on age category (18–29; 30–44; 45–54; 55–64; 65 or older), sex, year of entry into Sutter primary care, and primary-care follow-up time (0–6 months, 7–24 months, > 24 months).

### Migraine severity

Among cases (i.e., individuals with MPA > 10), we defined the following count variables to capture migraine severity (1) all-cause neurology visits per year; (2) migraine-specific urgent care visits per year; (3) triptans prescribed per year. We additionally defined two dichotomous measures to capture migraine severity: (4) 0 versus ≥1 migraine-specific emergency department (ED) visit during the study period; and (5) MPA score > 100 (more severe) versus MPA score 11–100 (less severe).

### Air pollution, methane emission, and oil and gas wells

We considered four separate exposure measures in our analyses. These included PM_2.5_, NO_2_, methane super-emitters, and active oil and gas wells. Exposure to air pollutants and to oil and gas wells was estimated based on average values at the beginning of the study period (in 2015). Methane emissions measures were based on data collected between 2016 and 2018.

#### PM_2.5_ and NO_2_

We used patient addresses to link annual average concentration of PM_2.5_ and NO_2_ estimates at the block group-level derived from annual-average integrated empirical geographic regression models [36]. The approach relied on universal kriging and took regulatory monitoring data, satellite imagery, and measures of land use and traffic as inputs. PM_2.5_ and NO_2_ achieved standardized RMSEs of 0.86 μg/m^3^ and 0.87 ppb, respectively. These variables were re-scaled such that coefficients in linear models correspond to each 5 μg/m^3^ increase in PM_2.5_ and each 5 ppb increase in NO_2_, respectively.

#### Methane emissions

Data on methane emissions were provided by CARB as described in Duren et al. 2019 [29]. In brief, CARB led the first California Methane Survey to provide systematic information on methane point sources across the state via Next Generation Airborne Visible/Infrared Imaging Spectrometer (AVIRIS-NG) flights conducted between 2016 and 2018. The AVIRIS-NG flights identified 564 distinct sources of methane plumes and captured average hourly emission rates in kilograms per hour (kg/hour). Examples of sources of methane plumes identified by AVIRIS-NG flights included oil and gas wells, dairies, and landfills. To estimate exposure to methane emissions for the present study, we calculated the sum kg/hour of emitted methane from all sources within 10 km of each participant *j*’s residence and weighted the emissions by the inverse-distance squared between each super-emitter, *i*, and patient *j*’s residence:

$$ {\sum}_{i=1}^n\frac{E_i}{d_{ij}^2}, $$

where *E* is the emission rate at super-emitter *i* in kg/hour and *d* is the distance in kilometers between super-emitter *i* and participant *j*. We created two exposure metrics based on methane emission rates. The first was the sum of methane emissions (in kg/hour) within 10 km, re-scaled so that model coefficients corresponded to a 100,000 kg/hour increase in methane emissions. The second was an indicator variable for presence of any methane super-emitter within 10 km.

#### Oil and gas wells

Finally, we obtained records for active oil and gas wells as of December 2015 from the California Division of Oil, Gas and Geothermal Resources website (CA DOGGR). To estimate exposure to active wells, we used inverse-distance weighting (IDW) of active wells within 10 km of each participant, *j*:

$$ {\sum}_{i=1}^n\frac{1}{d_{ij}^2}, $$

where *i* is an active well located within 10 km of the participant and *d* is the distance in kilometers between well *i* and participant *j*. We created two exposure metrics based on exposure to active wells. The first was a continuous IDW sum of all active wells within 10 km, re-scaled so that coefficients in linear models correspond to a 1000-unit increase in the IDW sum. The second was an indicator variable for presence of any active oil or gas well within 10 km.

### Statistical analyses

We first conducted a case-control analysis in which we examined the association between migraine status and each of the four exposures. Next, we conducted a case-case analysis to examine whether migraine severity was associated with each of the exposures.

#### Case-control analysis

For the case-control analyses, we used generalized linear mixed models with a logit link with county-specific random intercepts to account for potential within-county clustering. All models controlled for our matching variables: categorical age and sex, as recommended [37], and race/ethnicity, Medicaid use, number of primary care visits per year, and block group-level population density and poverty. We specified four separate statistical models to examine the association between migraine status and each of the environmental exposures of interest (i.e., PM_2.5_, NO_2_, methane super-emitters, and active oil and gas wells). We used generalized additive mixed models with penalized smoothing splines to capture potential non-linearities in the exposure-response relationships. As a secondary analysis, we used the binary exposure specification for both methane super-emitters and active wells (i.e., any super-emitter within 10 km vs. none and any well within 10 km vs. none).

#### Case-case analysis

In the case-case analyses, we utilized negative binomial mixed models (for count of neurology visits, migraine-related urgent care visits, and prescriptions for triptans) and logistic mixed models (for ≥1 migraine-related ED visit per person-year vs. less and MPA score > 100 vs. 10–100) with random intercepts for county to examine the association between migraine severity and the exposure of interest. We controlled for the same set of potential confounding variables as described for the case-control analysis, assessed deviations from linearity using penalized smoothing splines, and as a secondary analysis considered binary specifications of super-emitters and active wells.

#### Sensitivity analyses

We conducted the following sensitivity analyses. First, we separated exposure to super-emitters into two categories: (1) dairy/cattle manure and landfills and (2) all other industrial types, which included power plants, refineries, wastewater treatment facilities, oil and gas distribution (e.g., oil/gas compressors, gas distribution lines), and oil and gas production (e.g., oil/gas waste lagoons, oil/gas plugged wells). We did so under the assumption that methane co-pollutant emissions would differ by these two categories. Second, we repeated our main case-control and case-case analyses with additional adjustment for BMI category and marital status. Finally, in our case-case analysis of migraine-specific ED visits, we additionally adjusted for distance from the patient’s address of record to the nearest ED.

For all models, we evaluated residual spatial autocorrelation using Moran’s I [38], which indicated no residual spatial autocorrelation in any of the analyses. Analyses were conducted using R 3.6.0 (R Foundation for Statistical Computing, Vienna, Austria). The Columbia University (Protcol #: AAAT0085), University of California, Berkeley (Protocol #: 2013-10-5693), and Sutter Health (IRBNet #:1452543–1) Institutional Review Boards approved this study.

## Results

The study based included 1,433,236 individuals with at least one primary care visit within the Sutter Health system in Northern California between 2014 and 2018. Based on MPA score, we initially identified 92,673 migraine cases and 278,019 matched controls. We excluded 3065 cases and 7327 controls who resided outside of 27 Northern California counties; 29 cases and 100 controls who lacked block group-level poverty data; and 4 cases and 28 controls missing PM_2.5_ data (Supplementary Fig. 1). The final study population included 89,575 cases and 270,564 controls in 27 counties (Supplementary Fig. 2, Supplementary Fig. 3).

**Fig. 1:**
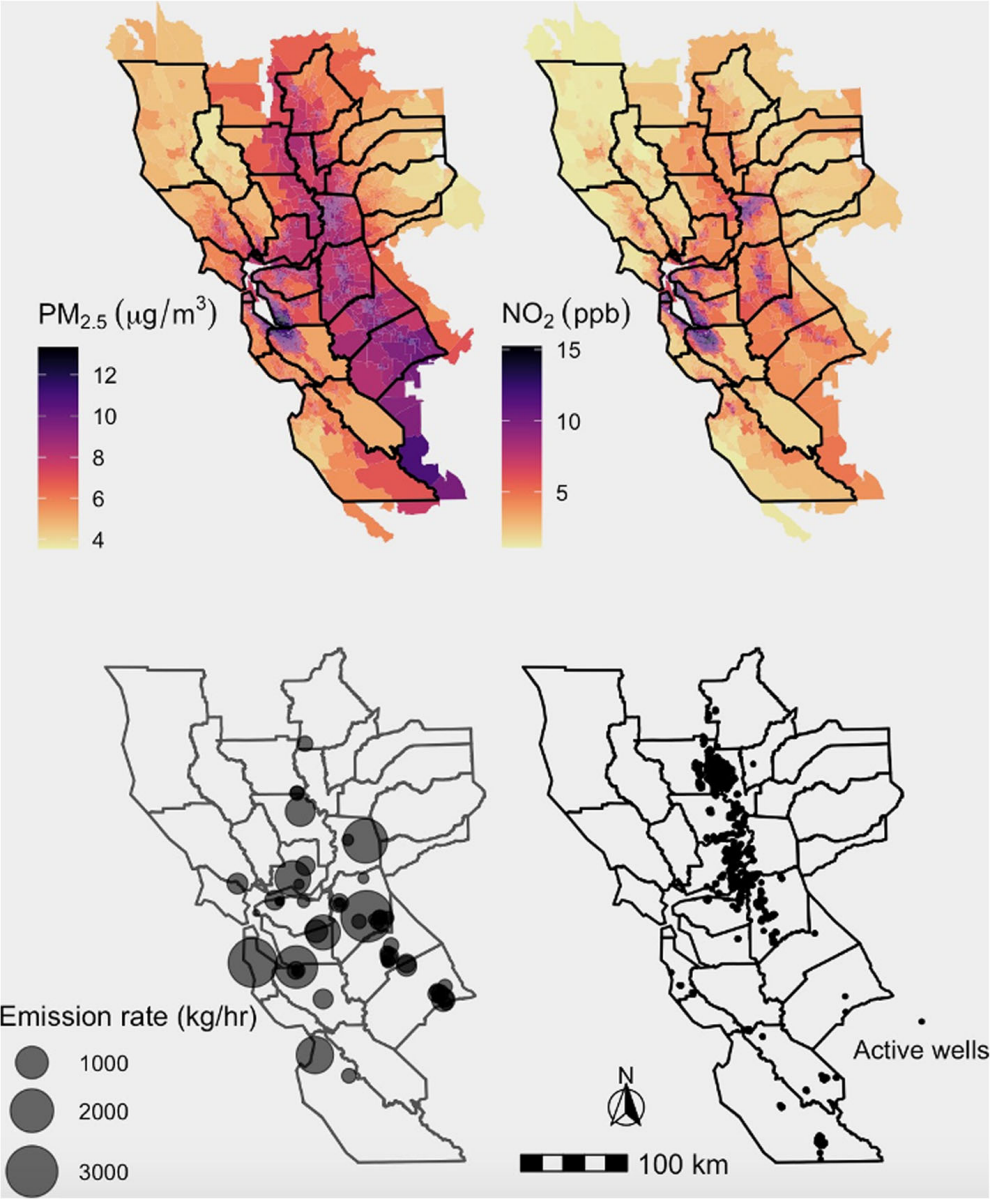
Distribution of environmental exposures within study region. Block group level 2015 annual average concentration of **a**. PM_2.5_ and **b**. NO_2_. **c**. Methane emission rate based on the California Methane Survey, conducted between 2016 and 2018. D. Location of active oil and gas wells as of December 2015

Migraine cases were most common between the ages of 30–44 years (*N* = 33,036, 36.9%) and occurred predominantly among females (*N* = 73,908, 82.5%). Migraine cases were more likely to be non-Hispanic white as compared with controls (58.7% versus 48.2%) and had more frequent primary care outpatient encounters and outpatient neurologist visits (Table 1). The 2015 average annual concentrations of PM_2.5_ and NO_2_; methane emission rates; and the location of active oil and gas wells are depicted in Fig. 1. The median PM_2.5_ concentration at patient addresses was 8.7 μg/m^3^ (min = 3.7, max = 13.3) and the median NO_2_ concentration was 7.7 ppb (min = 1.1, max = 15.2). Of 564 super-emitters surveyed in the state, 60 (10.6%) were located within 10 km of study participants, including 35 dairies/landfills and 25 other types of super-emitters.

**Table 1:** Patient demographics, healthcare utilization, and environmental exposures for migraine cases and controls from Sutter Health in Northern California, 2014–2018

	Migraine cases *N* = 89,575	Controls^**a**^ *N* = 270,564
**Patient Demographics**		
**Age Category**, N (%)		
18–29 years	16,952 (18.9)	51,112 (18.9)
30–44 years	33,036 (36.9)	99,792 (36.9)
45–54 years	19,226 (21.5)	58,169 (21.5)
55–64 years	12,578 (14.0)	38,093 (14.1)
≥ 65 years	7783 (8.7)	23,399 (8.7)
**Sex**, N (%)		
Female	73,908 (82.5)	223,230 (82.5)
Male	15,667 (17.5)	47,334 (17.5)
**Race/Ethnicity**, N (%)		
Non-Hispanic		
Asian	9278 (10.4)	52,794 (19.9)
Black	3685 (4.1)	10,253 (3.8)
White	52,579 (58.7)	130,418 (48.2)
Other	11,351 (12.7)	41,907 (15.5)
Hispanic	12,682 (14.2)	34,192 (12.6)
**Marital Status**, N (%)		
Divorced/Separated/Widowed	7444 (8.3)	18,881 (7.0)
Married/Significant Other	51,390 (57.4)	155,644 (57.5)
Single	22,659 (25.3)	63,801 (23.6)
Other/Unknown	8082 (9.0)	32,238 (11.9)
**Body Mass Index Category (kg/m**^**3**^**)**, N (%)		
Underweight (< 18.5)	1672 (1.9)	5636 (2.0)
Normal (18.5–24.9)	33,801 (37.7)	112,014 (41.4)
Overweight (25–29.9)	26,969 (30.1)	79,209 (29.3)
Obese Class 1 (30–34.9)	14,595 (16.3)	39,405 (14.6)
Obese Class 2 (35–39.9)	6835 (7.6)	17,388 (6.4)
Obese Class 3 (40+)	4614 (5.2)	11,658 (4.3)
Missing	1089 (1.2)	5254 (1.9)
**Block Group-Level Variables**, Median (IQR)		
Percent Poverty	7.2 (3.5, 14.3)	6.6 (3.2, 13.1)
Population Density (individuals per km^2^)	2211 (901, 3593)	2292 (954, 3592)
**Medicaid Beneficiary**, N (%)		
Yes	6929 (7.7)	15,105 (5.6)
No	82,646 (92.3)	255,459 (94.4)
**Healthcare Utilization**		
**Encounters per person-year**		
Primary care, Median (IQR)	2.4 (1.4, 4.0)	1.9 (1.2, 3.1)
Neurology, Mean (SD)	1.2 (3.4)	0.2 (1.0)
Urgent Care (Migraine-Specific), Mean (SD)	0.2 (2.7)	–
Emergency (Migraine-Specific), N (%)	0.1 (0.7)	–
≥ 1 visit during the study period	3987 (4.5)	–
< 1 visit during the study period	85,588 (95.5)	–
**Triptan prescriptions per person-year,** mean (SD)	0.6 (2.6)	
**MPA Score** – N (%)	66.6 (31.5)	–
11 - 100	59,599 (66.5)	–
> 100	29,976 (33.5)	–
**Environmental Exposures**		
**Air Pollutants,** Median (IQR)		
NO_2_, ppb	7.7 (5.7, 10.2)	8.1 (5.9, 10.4)
PM_2.5,_ μg/m^3^	8.7 (7.8, 9.6)	8.9 (7.8, 9.7)
**CH**_**4**_ **Emissions**		
Any super-emitter within 10 km, N (%)	18,457 (20.6)	57,224 (21.1)
Total IDW emissions in kg/hour, Mean (SD)	21,461 (192,973)	26,070 (180,548)
**Active Oil and Gas wells**		
Any oil or gas well within 10 km, N (%)	13,179 (14.7)	37,010 (13.7)
Total IDW wells, Mean (SD)	604 (6468)	603 (6459)

*IDW* Inverse-distance weighted; *IQR* Interquartile range; *MPA* Migraine probability algorithm^a^ Frequency-matched on age category, sex, year of entry into Sutter primary care, and primary-care follow-up time (0–6 months, 7–24 months, ≥ 24 months)

**Fig. 2:**
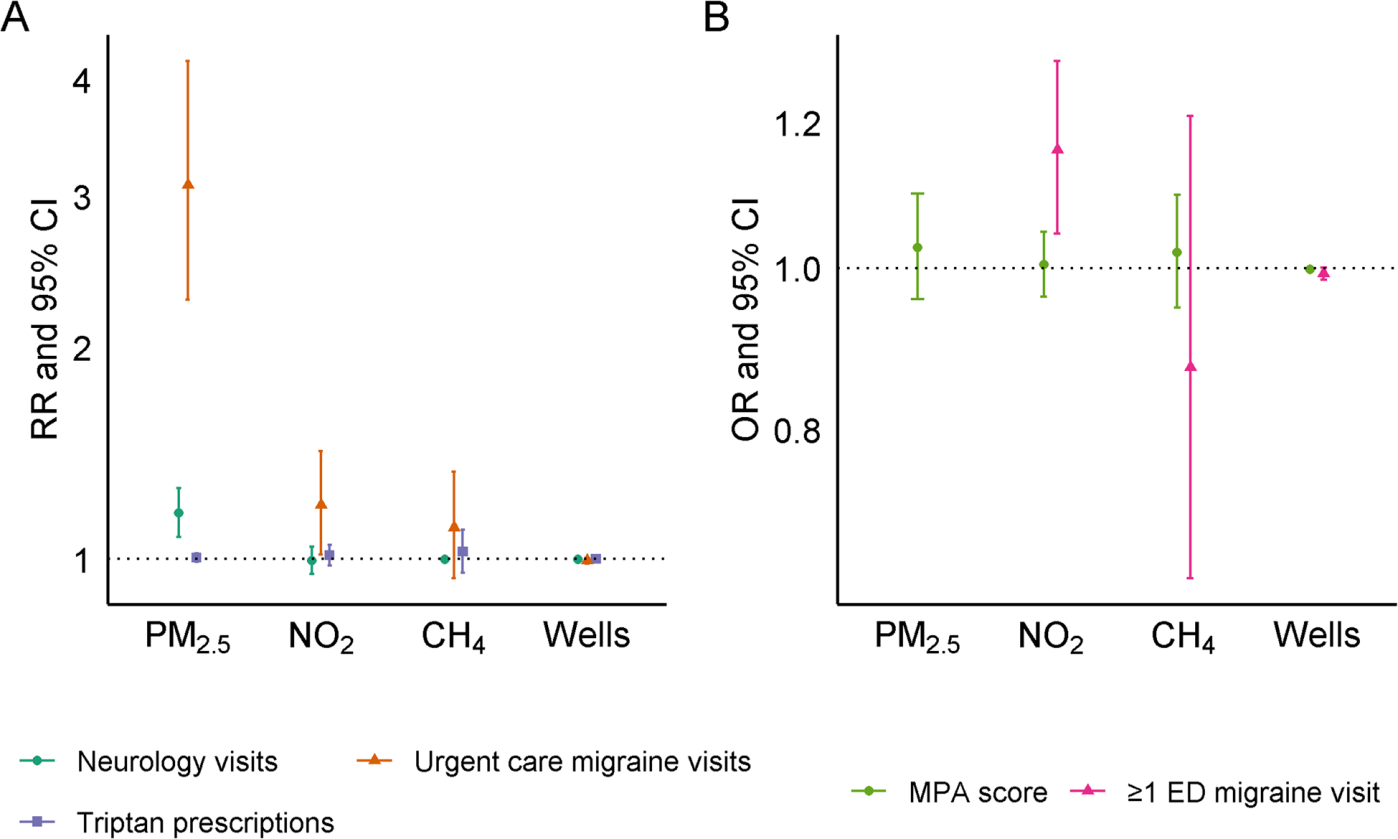
Association between environmental exposures and odds of being a migraine case versus control. Results from a mixed logistic model with a random intercept for county adjusted for individual-level age category (18–29, 30–44, 45–54, 55–64, ≥65), race/ethnicity (Hispanic, non-Hispanic Asian, non-Hispanic-Black, non-Hispanic White, and non-Hispanic other), sex, Medicaid use, number of primary care visits per person-year during the study period, and block group-level population density and poverty. OR are per 5 μg/m^3^ for PM_2.5_, per 5 ppb for NO_2_, per 100,000 kg/hour increase in IDW sum of methane emissions within 10 km for super-emitters, and per 1000-unit increase in IDW sum of all wells within 10 km for active oil and gas wells

In our case-control analysis we observed only linear associations between exposure and migraine case status. We found some evidence for an association between migraine case status and block-group level NO_2_ concentration. We estimated that for every 5 ppb increase in annual average NO_2_ concentration the odds of migraine case status increased by 1.02 times (95% CI: 1.00, 1.05). We also estimated that for every 100,000 kg/hour increase in IDW sum of methane emissions within 10 km, the odds of migraine case status also increased (OR = 1.04, 95% CI: 1.00, 1.08). We found no evidence of an association between migraine case status and block-group level PM_2.5_ concentrations or for active oil or gas wells within 10 km (Fig. 2**,** Supplementary Table 1A). In our secondary analysis with dichotomized methane emission and active wells, we found no association between any super-emitter or any active well within 10 km and migraine (Supplementary Table 1B).

In our case-case analysis, meant to evaluate the association between environmental exposures and migraine frequency/severity, we observed mostly linear relationships, except for the association between PM_2.5_ and odds of any migraine ED visit during the study period (Supplementary Fig. 4). For the other severity outcomes, we found that each 5 μg/m^3^ increase in annual average block-group level PM_2.5_ concentration was associated with increased frequency of outpatient neurology visits (RR = 1.18, 95% CI: 1.09, 1.29), increased frequency of migraine-specific urgent care visits (RR = 3.09, 95% CI: 2.28, 4.18) and MPA score greater than 100 (OR = 1.14, 95% CI: 1.07, 1.22). We found no evidence of an association between increased PM_2.5_ concentration and frequency of prescribed triptans (RR = 1.03, 95% CI: 0.97, 1.10).

**Fig. 3:**
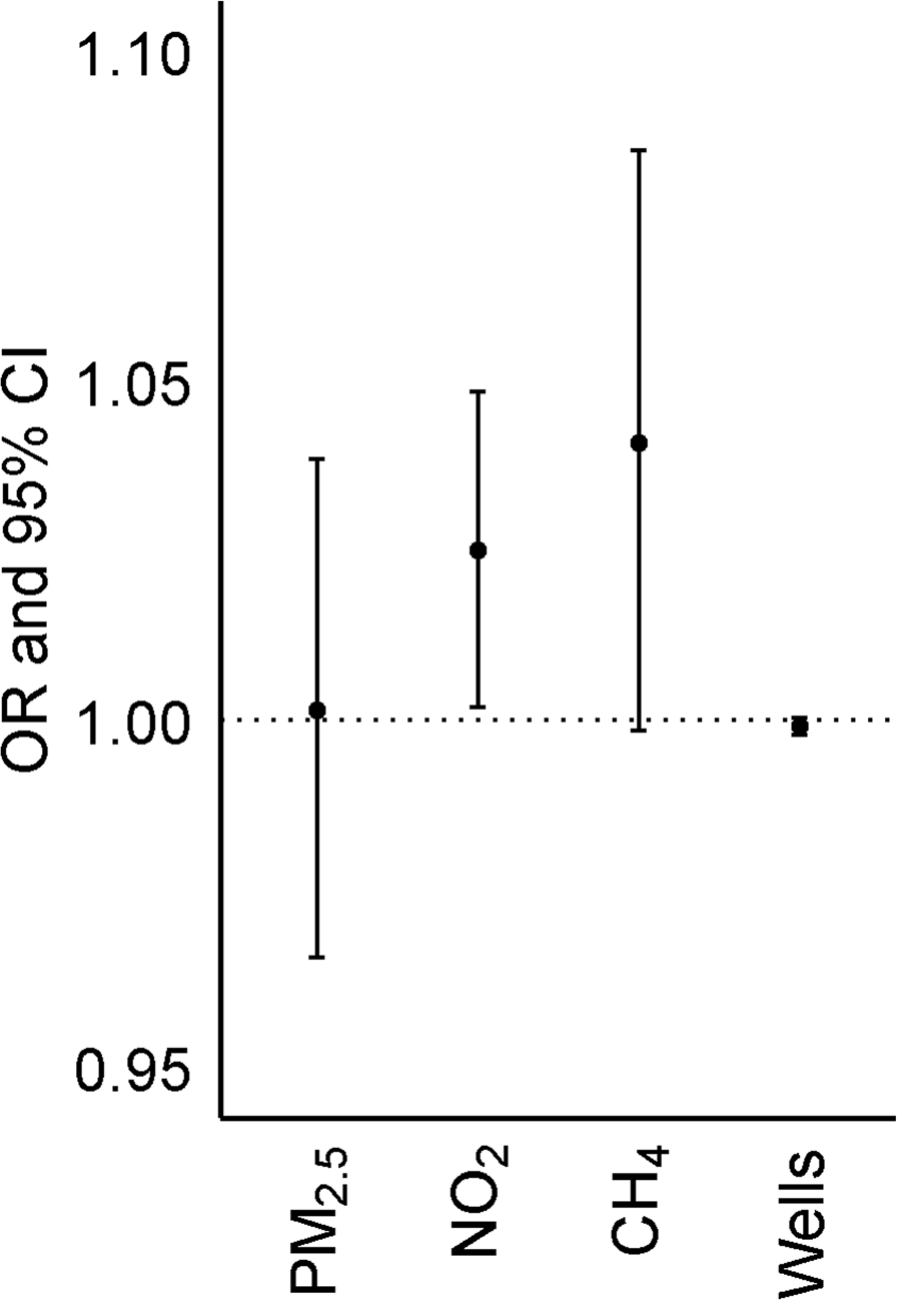
Association between environmental exposures and severity of migraine case status. Associations estimated with mixed logistic and negative binomial models with random intercepts for county adjusted for individual-level age category (18–29, 30–44, 45–54, 55–64, ≥65), race/ethnicity (Hispanic, non-Hispanic Asian, non-Hispanic-Black, non-Hispanic White, and non-Hispanic other), sex, Medicaid use, number of primary care visits per person-year during the study period, and block group-level population density and poverty. Neurology visits, urgent care migraine-specific visits, and triptan prescriptions were parameterized as continuous counts per person-year and analyzed using negative binomial models (Panel **a**). ED migraine visits were dichotomized as zero vs. ≥ 1 during the study period, and MPA score as > 100 versus less (Panel **b**). ORs and RRs are per 5 μg/m^3^ for PM_2.5_, per 5 ppb for NO_2_, per 100,000 kg/hour increase in IDW sum of methane emissions within 10 km for super-emitters, and per 1000-unit increase in IDW sum of all wells within 10 km for active oil and gas wells

Increased block group-level NO_2_ concentration was not associated with triptans, outpatient neurology visits or MPA score, but we found that each 5 ppb increase in NO_2_ concentration was associated with increased frequency of migraine-specific urgent care visits (RR = 1.22, 95% CI: 1.02, 1.46) and with increased odds of having at least one migraine-specific ED visit during follow-up (OR = 1.16, 95% CI: 1.05, 1.29) (Fig. 3, Supplementary Table 2A).

A 100,000-unit increase in the IDW sum of overall methane emissions within 10 km was associated with increased frequency of migraine-specific urgent care visits (RR = 1.12, 95% CI: 0.92, 1.36). Having any methane emitter within 10 km was also associated with increased frequency of urgent care visits (RR = 1.32, 95% CI: 1.14, 1.54) (Fig. 3, Supplementary Tables 2A and 2B). Proximity to super-emitters was not associated with the frequency of triptan prescriptions, outpatient neurologist visits, migraine-specific ED visits, or MPA score. Presence of any active oil and gas wells within 10 km was associated with increased frequency of outpatient neurologist visits (RR = 1.09, 95% CI: 1.03, 1.16), frequency of migraine-specific urgent care visits (RR = 1.43, 95% CI: 1.21, 1.70), and odds of at least one migraine-specific ED encounter per person-year of follow-up (OR = 1.11, 95% CI: 1.00, 1.24). We found no evidence of an association between our continuous measure of active oil and gas wells and any of the five measures of migraine severity (Fig. 3**,** Supplementary Tables 2A and 2B).

We conducted a sensitivity analysis in which we separately considered dairies and landfills versus all other methane super-emitters. Overall, these findings were largely consistent with our main findings for both the case-control and case-case analyses; the association was stronger for dairies and landfills (RR = 1.18, 95% CI: 0.37, 3.87) than for other super-emitters (1.08, 95% CI: 0.85, 1.36), albeit with widely overlapping confidence intervals. In re-analysis of the case-control and case-cases studies with additional controls for BMI category and marital status, results did not differ from those of our primary analysis (Supplementary Figs. 5 and 6). Results were also unchanged when we incorporated distance to the nearest Sutter hospital in the ED visit case-case analyses (Supplementary Fig. 7).

## Discussion

Past research links short-term exposure to a range of air pollutants with ED visits migraine headache. Our study builds upon previous studies and considers the implications of long-term environmental exposures for migraine. Using data from the Sutter Health EHR database in Northern California, we examined relationships between a wide range of environmental exposures–including PM_2.5_, NO_2_, methane super-emitters, and oil and gas wells–and both migraine headache and headache severity among patients with migraine. Our case-control analysis revealed increased odds of exposure to NO_2_ and methane super-emitters among patients with migraine as compared with frequency-matched population controls without clinical diagnosis of migraine. In our case-case analysis, migraine severity–as measured by frequency of triptan prescriptions, outpatient neurology visits, migraine-specific urgent care and ED visits, and MPA score–was most strongly and consistently associated with average PM_2.5_ and NO_2_ exposure.

Research to date has focused primarily on short-term exposure to air pollutants as a trigger for migraine. Although relatively few studies have focused on chronic exposure, evidence to date nevertheless suggests that chronic exposure to common pollutants may be important in the etiology, severity, or frequency of headache including migraine. Using linked records from the Taiwan National Health Insurance Research Database and Taiwan Air Quality Monitoring Database, Hong et al. (2020) found that frequency of recurrent headaches among children younger than 18 years of age increased with higher-level exposure to several air pollutants including PM_2.5_, CH_4_, NO_2_, and total hydrocarbons [39]. Adetona et al. (2020) conducted a cross-sectional study among residents of a community adjacent to a large open landfill in Lagos, Nigeria. Results of that study indicated that chronic exposure to emissions from open combustion of municipal solid waste—a major source of particulate matter, polycyclic aromatic hydrocarbons, and toxicants such as polychlorinated biphenyls and brominated flame retardants—was associated with increased odds of daily occurrence of headache [40]. Moreover, in animal models, chronic exposure to acrolein, which is prevalent in both indoor and outdoor air pollution, yielded physiologic changes consistent with migraine [41, 42].

Results of the present study further demonstrate the potential importance of long-term residential exposures for migraine severity. One important implication of these findings is that in more heavily polluted communities, individuals may be more likely to suffer from migraines or may suffer from more frequent headaches. The existing literature consistently demonstrates the disproportionate burden of air pollution in already disadvantaged communities [43–45], and the substantial economic and social costs associated with migraine in the United States [2, 46–49]. Our findings therefore motivate careful examination of the extent to which disparate levels of exposure to harmful emissions and levels of community air pollution translate to greater burden of migraine headache and the associated economic and social costs particularly in already disadvantaged communities.

To our knowledge, ours is the first study to examine the implications of exposure to methane super-emitters for migraine; we identified an association between super-emitter exposure and migraine case status but not migraine severity. Methane super-emitters included dairies and waste lagoons, landfills, power plants, refineries, wastewater treatment facilities, and oil and gas production and distribution infrastructure. Although methane itself is not directly toxic to humans, it is often co-emitted with other noxious compounds. The heterogeneous group of super-emitters considered in this study also produce a wide range of co-pollutants including volatile organic compounds, ammonia, hydrogen sulfide, and particulate matter, several of which are odorous [50–52]. Methane also contributes to the formation of ground-level ozone, previously implicated as a trigger for migraine headache [23, 24]. In addition, super-emitters, such as oil and gas wells, produce noise pollution [53]. Both noise and odors have been consistently linked with migraine headache [8, 13–15].

Importantly, we assigned methane super-emitter exposure based on data collected between 2016 and 2018, while we included migraine cases in the Sutter EHR database between 2014 and 2018. This complicates the temporal ordering of exposure and response. However, reverse causality seems an implausible alternative explanation for our results, as we know of no reason that individuals with migraine would cause systematic increases in local super-emitter exposure or would move closer to a super-emitter post-diagnosis. It is possible, however, that our findings reflect residential sorting of individuals predisposed to migraine into localities where methane emissions are higher on average [54, 55]. In the U.S., migraine follows a social gradient and is more common among lower-income individuals who are also more likely to live in more polluted neighborhoods [2, 3]. We aimed to address this important source of confounding by adjusting for patient Medicaid use and block-group-level population density and poverty. Future research should specifically examine co-pollutants that may explain the apparent link between methane emissions and migraine, and to disentangle the role of residential sorting and confounding by socioeconomic status from any etiologic role that methane plays in the onset or exacerbation of migraine headaches.

Unlike several prior studies that rely on ED visits as a rough proxy for disease severity, our case-case analysis considered a more comprehensive set of proxies obtained from EHR data including non-emergency migraine-specific healthcare visits, migraine-related medication use, a validated migraine severity score, and overall neurology visits among patients with migraine. We also used splines to consider potential non-linearities in exposure-response relationships between each environmental exposure and our migraine severity outcome measures. Consistent with past research [2, 3], we observed an association between NO_2_ exposure and migraine severity as measured by migraine-specific urgent care visits and migraine-specific ED visits even at NO_2_ levels well below the current national standards (our population-average annual exposure was around 8 ppb compared to the U.S. Environmental Protection Agency annual standard of 53 ppb).

Past research finds an association between short-term exposure to PM_2.5_ and migraine-specific ED visits [2, 3]. Our analysis demonstrated an association with long-term, annual average PM_2.5_ across a more comprehensive set of clinical proxies for headache severity, including outpatient neurology visits and migraine-specific urgent care visits. For ED visits, we found a paradoxical inverse u-shaped exposure-response wherein individuals with the lowest and highest levels of average PM_2.5_ had the lowest odds of ED visit. This relationship persisted even after we incorporated additional statistical controls for distance to nearest Sutter ED. As our analysis differs from previous studies that consider short-term PM_2.5_ levels and risk of ED visits, this finding could reflect misalignment of the examined exposure window (annual average PM_2.5_) with an acute outcome (ED visits).

Communities with higher annual PM_2.5_ concentrations may also have higher peak and long-term average exposure that gives rise to ED visits. We know of no research that demonstrates higher levels of PM_2.5_ as protective against migraine headaches. This relationship could reflect residential sorting where individuals with migraine move out of high PM_2.5_ communities. As migraine-specific emergency department visits are relatively rare in these data, we suspect that the observed relationship is driven by relatively less frequent use of emergency departments for headache among individuals living in the few counties with the highest PM_2.5_ levels. This finding also implies possible geographic disparities in either access to or use of care for severe migraine headaches unrelated to proximity or insurance status that should be explored in future research.

The association between PM_2.5_ and migraine severity may be partly explained by correlation between PM_2.5_ and other exposures known to precipitate migraine headache (namely, noise and noxious odors) [8, 13–15]. PM_2.5_, is known to activate the sympathetic nervous system, result in systemic inflammation, and trigger cardiovascular events [56, 57], and may also directly result in migraine. The smallest fraction of the PM_2.5_ particles, ultrafine particulate matter (≤ 0.1 μm in diameter [58]), may have a disproportionately large role. Ultrafine particles–unlike the larger component particles of PM_2.5_–can transverse the blood-brain barrier and reach the brain directly through the olfactory bulb [59].

Despite making up just a small portion of the total PM_2.5_ mass concentration, these circumstances raise the possibility that the apparent association between PM_2.5_ and migraine severity in this and previous studies could be partially explained by neurotoxic effects secondary to exposure to the ultrafine component of PM_2.5_ [60, 61]. The U.S. EPA does not regulate ultrafine particulate matter, meaning exposure estimates are sparse and epidemiologic studies rare. Future migraine research should aim to evaluate the effects of ultrafine particles on migraine and disentangle the effects of concomitant exposure to noise, odor, PM_2.5_, and ultrafine particles.

Our analyses include all individuals with migraine followed from 2014 to 2018 but do not distinguish between individuals with previously diagnosed migraine at the beginning of the study period (i.e., prevalent cases) and individuals diagnosed with migraine throughout the study period (i.e., incident cases). This makes ascertainment of an etiologic role of environmental exposures in either migraine onset or exacerbation challenging. As discussed previously, we cannot eliminate the possibility that our findings may reflect residential sorting, wherein individuals with existing migraine are more likely to reside in health-harming communities, for example those of lower socioeconomic status or with higher levels of pollutants. Alternatively, individuals with migraine and the financial means to do so may choose to leave communities with environmental exposures that trigger their headaches. The direction and magnitude of bias attributable to residential sorting is therefore difficult to anticipate.

Although our analyses include individuals with migraine followed from 2014 to 2018, exposures were either measured at the beginning of the study period in 2015 (annual average PM_2.5_, NO_2_, and presence of oil and gas wells) or as values between 2016 and 2018 (methane super-emitter emissions and presence). We assume relatively stable levels of long-term air pollution and oil and gas well exposure during the study period. Methane super-emitter measurements took place between 2016 and 2018, but emission trends likely vary over time. Exposures were also assigned based on a single residential address on the index date and therefore do not capture exposure accrued during time spent outside the home and also do not reflect potential moves between 2014 and 2018. Future research should endeavor to incorporate time-varying measures of air pollution, oil and gas wells, and methane emissions in relation to migraine onset and exacerbation in order to better characterize the dynamic relationship between environment and migraine.

Residential addresses were ascertained in October 2019 after the study period. Selection bias could result, for example, if individuals with migraine headache in highly polluted counties moved to less polluted counties *outside* of the Sutter catchment areas. Because a small minority of individuals lived outside of the Sutter catchment area in October 2019 (3.3% of cases and 2.6% of controls), we expect any resultant bias to be minimal. Some differential exposure misclassification could also arise if individuals with migraine headache in highly polluted counties moved to less polluted counties *within* the Sutter catchment area, leading to systematic underestimation of long-term exposures among cases, and therefore, underestimation of effect estimates.

While our study incorporates a more comprehensive set of proxy measures for migraine headaches as compared with previous studies (which typically relied on migraine ED visits), we lacked any direct measure of headache frequency among patients with migraine (e.g., headache diaries). Our results rest on patients seeking clinical care for migraine. If individuals with higher levels of environmental exposure were systematically less likely to seek migraine treatment, our results may be attenuated. Headache diaries would circumvent this problem and further examination of the relationship between migraine and the environment in datasets where direct measures of headache frequency are available [62–64] would further our understanding of this relationship.

Fourth, our analysis includes a comprehensive set of potential confounding variables. Nevertheless, we note the absence of several critical variables–including individual-level income, educational attainment, and employment status–that may be important confounders in studies that use treatment seeking as a proxy for headache severity, given past research showing that migraine plays a key role in disability, absence from work or school, and that migraine follows a social gradient and is less common in wealthier individuals [2, 5, 7]. Further, we lacked information on environmental noise pollution, which may trigger migraines [65] and often co-occurs with sources of air pollution.

Finally, we drew participants from a single healthcare system in Northern California. This may limit generalizability to other populations including individuals who are uninsured or have limited health insurance. Northern California also differs meaningfully from the rest of the U.S. in the quality and extent of environmental exposures and population demographics. The relationship between migraine and environment may differ by region, season, and based on individual characteristics. This motivates ongoing study of the relationship between migraine in the environment in varied contexts.

## Conclusions

In this study, we demonstrate an association between long-term NO_2_ and methane super-emitter exposure and odds of being a migraine patient. We also find annual average NO_2_ and PM_2.5_ exposure associated with migraine headache severity. Our study expanded the scope of environmental pollutants considered as risk factors for migraine and included numerous measures of migraine severity derived from EHR data and contributes to the existing literature on migraine and the environment by explicitly considering long-term exposure to common pollutants. These findings illustrate the potential role of ambient air pollution for prevalent migraine and migraine severity. Future studies are needed that establish the temporal ordering of exposure and outcome and the relevant exposure period as well as that determine the most relevant air pollutants. In addition, researchers should consider the potential heterogeneity in the relationship between migraine and the environment across different geographic contexts and within population subgroups. Such studies could identify environmental risk factors on which we could intervene to reduce the population burden of migraine.

## Supplementary Information

**Additional file 1: Fig. S1**. Ascertainment of migraine cases and controls from Sutter Health electronic health record data, 2015–2018. **Fig. S2.** Counties included in the analysis in Northern California (left) and distribution of Sutter hospitals (right). **Fig. S3.** Distribution of migraine cases and controls. **Fig. S4.** Flexible dose-response between levels of PM_2.5_ (μg/m3) and odds of having ≥1 ED visit over the course of the study period. From mixed logistic models with penalized smoothing splines for PM2.5, random intercept for county, adjusted for individual-level age category (18–29, 30–44, 45–54, 55–64, ^3^ 65), race/ethnicity (Hispanic, non-Hispanic Asian, non-Hispanic-Black, non-Hispanic White, and non-Hispanic other), sex, Medicaid use, number of primary care visits per person-year during the study period, and block group-level population density and poverty. **Fig. S5.** Association between environmental exposures and odds of being a migraine case versus control. **Fig. S6.** Association between environmental exposures and severity of migraine case status. **Fig. S7.** Association between PM2.5 and migraine-specific ED visits, adjusted for distance to nearest Sutter hospital. **Table S1A.** Associations between continuous environmental exposures and migraine status. **Table S1B.** Associations between dichotomized environmental exposures and migraine status. **Table S2A.** Associations between continuous environmental exposures and measures of migraine severity. **Table S2B.** Associations between binary environmental exposures and measures of migraine severity.

## Data Availability

The Sutter Health electronic health record data are considered Protected Health Information under the Health Insurance Portability and Accountability Act of 1996 (HIPAA) in the United States, and as such are not publicly-available. PM_2.5_ and NO_2_ data are available for download at: https://www.caces.us/data. Methane data are available via https://www.nature.com/articles/s41586-019-1720-3#data-availability. Oil and gas well data are available at https://www.conservation.ca.gov/calgem/Pages/Oil-and-Gas.aspx.

## Abbreviations

BMI: Body mass index
CARB: California air resources board
ED: Emergency department
EHR: Electronic health records
IDW: Inverse distance weighted
MPA: Migraine probability algorithm
NO_2_: Nitrogen dioxide
PM_2.5_: Fine particulate matter
SO_2_: Sulfur dioxide
U.S.: United States
VOCs: Volatile organic compounds
