# Correction to: Air pollution, methane super-emitters, and oil and gas wells in Northern California: the relationship with migraine headache prevalence and exacerbation

**DOI:** 10.1186/s12940-021-00745-8

**Published:** 2021-05-10

**Authors:** Holly Elser, Rachel Morello-Frosch, Alice Jacobson, Alice Pressman, Marianthi-Anna Kioumourtzoglou, Richard Reimer, Joan A. Casey

**Affiliations:** 1 Stanford University School of Medicine, Stanford Center for Population Health Sciences, Stanford, USA; 2 Department of Environmental Science, Policy, and Management and School of Public Health, University of California Berkeley, Berkeley, CA USA; 3 Research, Development and Dissemination, Sutter Health, Sacramento, USA; 4 Department of Environmental Health Sciences, Mailman School of Public Health, Columbia University, 722 W 168th St, Rm 1206, New York, NY 10032-3727 USA; 5 Department of Neurology and Neurological Science, Stanford University School of Medicine, Stanford, USA

**Correction to: Environ Health 20, 45 (2021)**

**https://doi.org/10.1186/s12940-021-00727-w**

Following the publication of the original article [1], it was noted that due to a typesetting error the actual figures for Figures 2 and 3 were interchanged.

The original article has been updated.
