# Supplementary material for: Air pollution, methane super-emitters, and oil and gas wells in Northern California: the relationship with migraine headache prevalence and exacerbation
Source: Environ Health. 2021 Apr 17;20:45. doi: 10.1186/s12940-021-00727-w (PMC8053292; doi:10.1186/s12940-021-00727-w)
Supplement: Supplementary file 1 — Additional file 1: Fig. S1. Ascertainment of migraine cases and controls from Sutter Health electronic health record data, 2015–2018. Fig. S2. Counties included in the analysis in Northern California (left) and distribution of Sutter hospitals (right). Fig. S3. Distribution of migraine cases and controls. Fig. S4. Flexible dose-response between levels of PM2.5 (μg/m3) and odds of having ≥1 ED visit over the course of the study period. From mixed logistic models with penalized smoothing splines for PM2.5, random intercept for county, adjusted for individual-level age category (18–29, 30–44, 45–54, 55–64, 3 65), race/ethnicity (Hispanic, non-Hispanic Asian, non-Hispanic-Black, non-Hispanic White, and non-Hispanic other), sex, Medicaid use, number of primary care visits per person-year during the study period, and block group-level population density and poverty. Fig. S5. Association between environmental exposures and odds of being a migraine case versus control. Fig. S6. Association between environmental exposures and severity of migraine case status. Fig. S7. Association between PM2.5 and migraine-specific ED visits, adjusted for distance to nearest Sutter hospital. Table S1A. Associations between continuous environmental exposures and migraine status. Table S1B. Associations between dichotomized environmental exposures and migraine status. Table S2A. Associations between continuous environmental exposures and measures of migraine severity. Table S2B. Associations between binary environmental exposures and measures of migraine severity. [file 12940_2021_727_MOESM1_ESM.pdf]

Supplementary Information for: Air pollution, methane super-emitters, and oil and gas wells in Northern California: the relationship with migraine headache prevalence and exacerbation

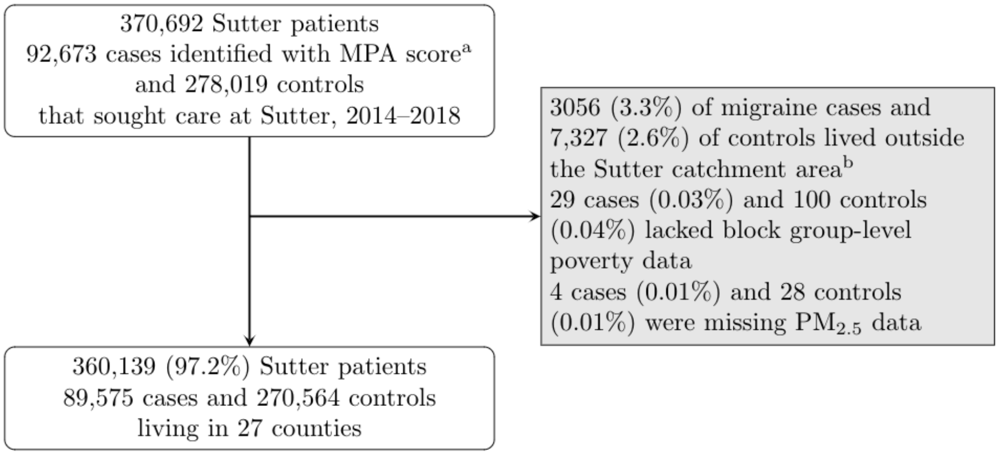

Supplementary Figure 1: **Ascertainment of migraine cases and controls from Sutter Health electronic health record data, 2015–2018.**

[a] The migraine probability algorithm (MPA) is based on migraine-related *International Classification of Diseases*-9 and 10 (*ICD*-9 and *ICD*-10) codes in the primary or secondary position in the outpatient or emergency department setting, on the patient's Significant Health Problem List, migraine prescription medications, and outpatient *ICD* codes related to cluster headache.

[b] Catchment counties include Alameda County, Amador County, Butte County, Colusa County, Contra Costa County, El Dorado County, Lake County, Mendocino County, Monterey County, Napa County, Marin County, Merced County, Nevada County, Placer County, Sacramento County, San Benito County, San Francisco County, San Joaquin County, San Mateo County, Santa Clara County, Santa Cruz County, Solano County, Sonoma County, Stanislaus County, Sutter County, Yolo County, Yuba County.

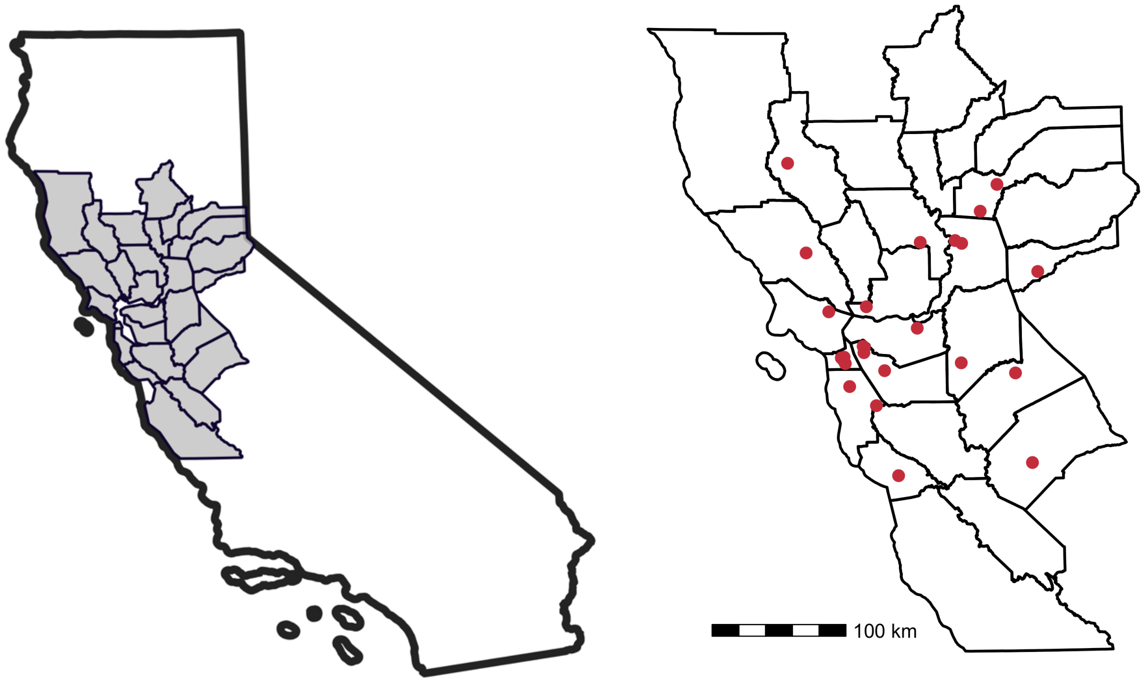

Supplementary Figure 2. Counties included in the analysis in Northern California (left) and distribution of Sutter hospitals (right).

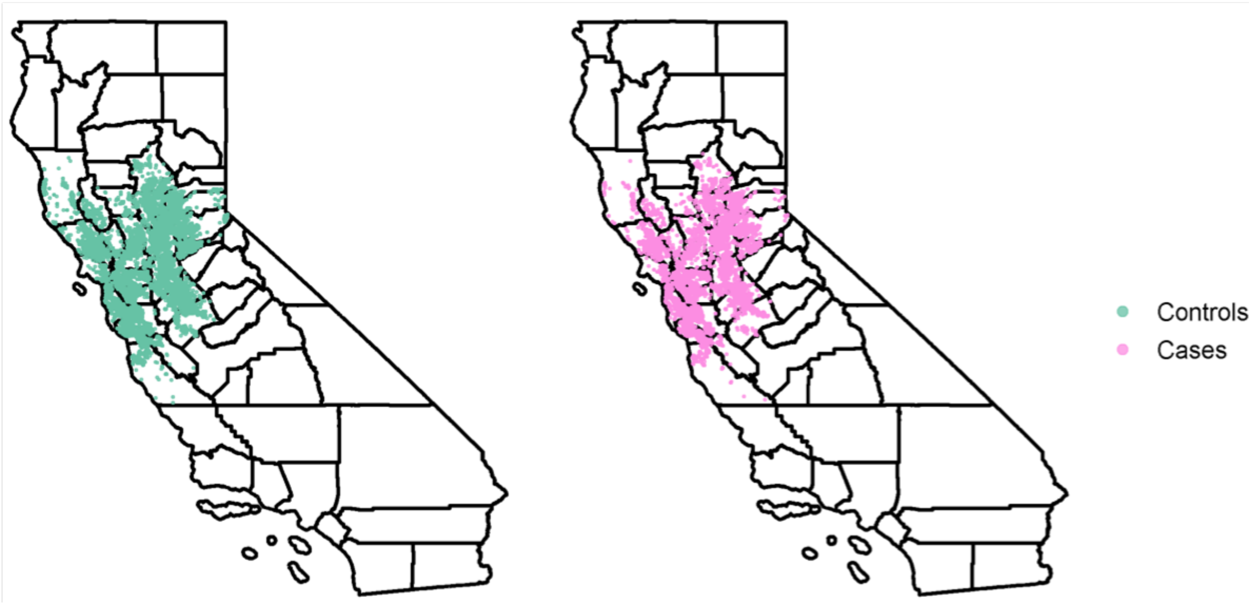

Supplementary Figure 3: Distribution of migraine cases and controls.

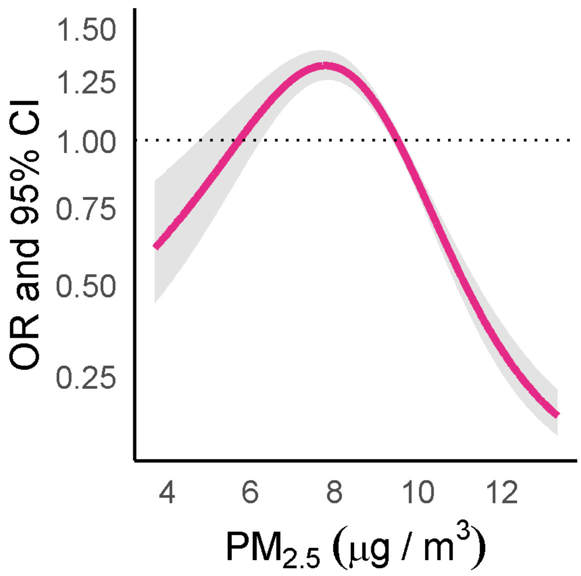

Supplementary Figure 4. Flexible dose-response between levels of $PM_{2.5}$ (μg/m^3) and odds of having ≥ 1 ED visit over the course of the study period. From mixed logistic models with penalized smoothing splines for $PM_{2.5}$, random intercept for county, adjusted for individual-level age category (18-29, 30-44, 45-54, 55-64, ≥ 65), race/ethnicity (Hispanic, non-Hispanic Asian, non-Hispanic-Black, non-Hispanic White, and non-Hispanic other), sex, Medicaid use, number of primary care visits per person-year during the study period, and block group-level population density and poverty.

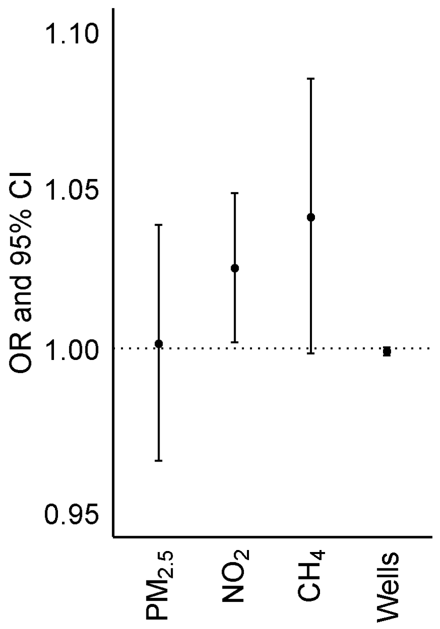

Supplemental Figure 5: Association between environmental exposures and odds of being a migraine case versus control. Results from a mixed logistic model with a random intercept for county adjusted for BMI category (underweight < 18.5; normal weight 18.5 – 24.9; overweight 25 – 29.9; obese class I 30 – 34.9; obese class 2 30 – 34.9; obese class 3 40+; missing), marital status (divorced, separated widowed; married or significant other; single; other or unknown), individual-level age category (18-29, 30-44, 45-54, 55-64, ≥65), race/ethnicity (Hispanic, non-Hispanic Asian, non-Hispanic-Black, non-Hispanic White, and non-Hispanic other), sex, Medicaid use, number of primary care visits per person-year during the study period, and block group-level population density and poverty. OR are per 5μg/m^3 for $PM_{2.5}$, per 5ppb for NO_2, per 100,000 kg/hour increase in IDW sum of methane emissions within 10km for super-emitters, and per 1,000-unit increase in IDW sum of all wells within 10km for active oil and gas wells.

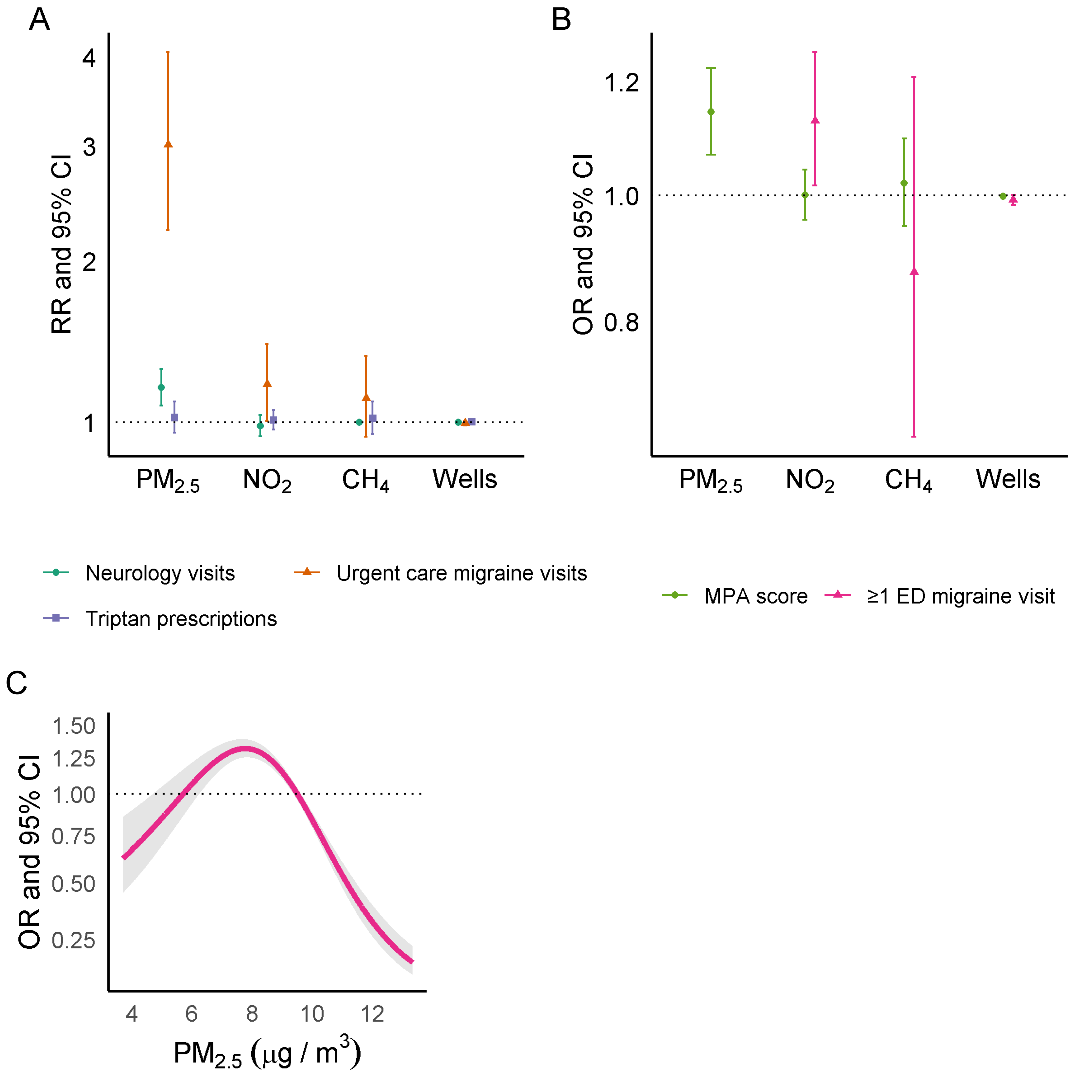

Supplemental Figure 6. Association between environmental exposures and severity of migraine case status. Associations estimated with mixed logistic and negative binomial models with random intercepts for county adjusted for BMI category (underweight < 18.5; normal weight 18.5 – 24.9; overweight 25 – 29.9; obese class I 30 – 34.9; obese class 2 30 – 34.9; obese class 3 40+; missing), marital status (divorced, separated widowed; married or significant other; single; other or unknown), individual-level age category (18-29, 30-44, 45-54, 55-64, ≥65), race/ethnicity (Hispanic, non-Hispanic Asian, non-Hispanic-Black, non-Hispanic White, and non-Hispanic other), sex, Medicaid use, number of primary care visits per person-year during the study period, and block group-level population density and poverty. Neurology visits, urgent care migraine-specific visits, and triptan prescriptions were parameterized as continuous counts per person-year and analyzed using negative binomial models (**Panel A**). ED migraine visits were dichotomized as zero versus ≥ 1 during the study period, and MPA score as >100 versus less. ORs and RRs are per 5μg/m^3 for $PM_{2.5}$, per 5ppb for NO_2, per 100,000 kg/hour increase in IDW sum of methane emissions within 10km for super-emitters, and per 1,000-unit increase in IDW sum of all wells within 10km for active oil and gas wells (**Panel B**). Non-linear exposure response curve for the association between levels of $PM_{2.5}$ (μg/m^3) and odds of having ≥ 1 ED visit over the course of the study period was modeled using mixed logistic models with penalized smoothing splines for $PM_{2.5}$ (**Panel C**).

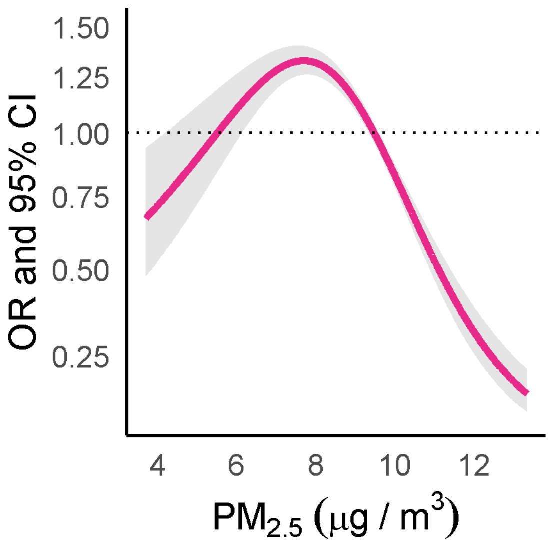

Supplementary Figure 7. Association between $PM_{2.5}$ and migraine-specific ED visits, adjusted for distance to nearest Sutter hospital. Association estimated with a mixed logistic models with penalized smoothing splines for $PM_{2.5}$ and a random intercept for county adjusted for individual-level age category (18-29, 30-44, 45-54, 55-64, ≥65), race/ethnicity (Hispanic, non-Hispanic Asian, non-Hispanic-Black, non-Hispanic White, and non-Hispanic other), sex, Medicaid use, number of primary care visits per person-year during the study period, distance to nearest Sutter hospital in kilometers, and block group-level population density and poverty.

Supplementary Table 1A. Associations between continuous environmental exposures and migraine status

	**Odds Ratio (95% CI)[a]**
**$PM_{2.5}$[b]**	1.00 (0.97, 1.04)
**NO_2[c]**	1.02 (1.00, 1.05)
**Methane Super-emitters[d]**	1.04 (1.00, 1.08)
Overall	1.04 (1.00, 1.08)
Dairies and Landfills[e]	1.07 (0.83, 1.39)
Other Super-emitters[f]	1.05 (1.00, 1.10)
**Active Oil & Gas Wells[g]**	0.99 (0.99, 1.00)

a. From a mixed logistic model with a random intercept for county, adjusted for individual-level age category (18-29, 30-44, 45-54, 55-64, 65 or older), race/ethnicity (Hispanic, non-Hispanic Asian, non-Hispanic-Black, non-Hispanic White, and non-Hispanic other), sex, Medicaid use, number of primary care visits per person-year during the study period, and block group-level population density and poverty.
b. OR corresponds to a 5µg/m^3 increase in levels $PM_{2.5}$.
c. OR corresponds to a 5ppb increase in levels of NO_2.
d. OR corresponds to a 100,000 kg/hr increase in IDW sum of methane emissions within 10km.
e. Includes dairy/livestock manure, landfills, compost.
f. Includes powerplants, refineries, wastewater treatment facilities, oil and gas distribution (e.g., oil/gas compressors).
g. OR corresponds to a 1,000-unit increase in IDW sum of wells within 10km.

Supplementary Table 1B. Associations between dichotomized environmental exposures and migraine status

	**Odds Ratio (95% CI)[a]**
**Methane Super-emitters[b]**	1.01 (0.99, 1.04)
**Active Oil & Gas Wells[c]**	1.01 (0.98, 1.04)

a. From a mixed logistic model with a random intercept for county, adjusted for individual-level age category (18-29, 30-44, 45-54, 55-64, ≥ 65), race/ethnicity (Hispanic, non-Hispanic Asian, non-Hispanic-Black, non-Hispanic White, and non-Hispanic other), sex, Medicaid use, number of primary care visits per person-year during the study period, and block group-level population density and poverty.
b. OR compares any methane super-emitter within 10km versus none.
c. OR compares any active oil & gas wells within 10km versus none.

Supplementary Table 2A. Associations between continuous environmental exposures and measures of migraine severity

	Measures of Migraine Severity				
	**Triptans**[a] RR (95% CI)	**Neurology Visit**[a] RR (95% CI)	**Urgent Care Visit**[a] RR (95% CI)	**ED Visits**[b] OR (95% CI)	**MPA Score**[b] OR (95% CI)
**$PM_{2.5}$**[c]	1.01 (0.99, 1.02)	1.18 (1.09, 1.29)	3.09 (2.28, 4.18)	Non-Linear	1.14 (1.07, 1.22)
**NO_2**[d]	1.01 (0.98, 1.06)	0.99 (0.94, 1.05)	1.22 (1.02, 1.46)	1.16 (1.05, 1.29)	1.00 (0.96, 1.05)
**Methane Super-emitters**[e]	1.03 (0.95, 1.12)	0.95 (0.85, 1.05)	1.12 (0.92, 1.36)	0.88 (0.63, 1.21)	1.01 (0.94, 1.09)
Overall	1.03 (0.95, 1.12)	0.95 (0.85, 1.05)	1.12 (0.92, 1.36)	0.88 (0.63, 1.21)	1.01 (0.94, 1.09)
Dairies and Landfills[f]	0.25 (0.02, 2.67)	0.91 (0.48, 1.71)	1.18 (0.36, 3.87)	0.96 (0.35, 2.68)	1.03 (0.67, 1.60)
Other Super-emitters[g]	1.02 (0.93, 1.12)	0.91 (0.79, 1.05)	1.08 (0.85, 1.36)	0.94 (0.74, 1.24)	1.03 (0.95, 1.12)
**Active Oil & Gas Wells**[h]	1.00 (1.00, 1.01)	1.00 (1.00, 1.00)	0.99 (0.98, 1.01)	0.99 (0.98, 1.01)	0.99 (0.99, 1.00)

a. From mixed negative binomial models for frequency of triptans, neurology visits, and migraine-specific urgent care visits. All models included a random intercept for county, adjusted for individual-level age category (18-29, 30-44, 45-54, 55-64, ≥ 65), race/ethnicity (Hispanic, non-Hispanic Asian, non-Hispanic-Black, non-Hispanic White, and non-Hispanic other), sex, Medicaid use, number of primary care visits per person-year during the study period, and block group-level population density and poverty.

b. From mixed logistic models for ≥ 1 ED migraine visit during the study period and MPA score > 100. All models included a random intercept for county, adjusted for individual-level age category (18-29, 30-44, 45-54, 55-64, ≥ 65), race/ethnicity (Hispanic, non-Hispanic Asian, non-Hispanic-Black, non-Hispanic White, and non-Hispanic other), sex, Medicaid use, number of primary care visits per person-year during the study period, and block group-level population density and poverty.

c. Coefficients corresponds to a 5$\mu g/m^3$ increase in levels $PM_{2.5}$.

d. Coefficients corresponds to a 5ppb increase in levels of NO_2.

e. Coefficients corresponds to a 100,000 kg/hr increase in IDW sum of CH_4 emissions within 10km.

f. Includes dairy/livestock manure, landfills, and compost.

g. Includes powerplants, refineries, wastewater treatment facilities, oil and gas distribution (e.g., oil/gas compressors, gas distribution lines), oil and gas production (e.g., oil/gas waste lagoons, oil/gas plugged wells.

h. Coefficients corresponds to 1,000-unit increase in IDW sum of wells within 10km.

Supplementary Table 2B. Associations between binary environmental exposures and measures of migraine severity

	Measures of Migraine Severity				
	**Triptans**[a] RR (95% CI)	**Neurology Visit**[a] RR (95% CI)	**Urgent Care Visit**[a] RR (95% CI)	**ED Visits**[b] OR (95% CI)	**MPA Score**[b] OR (95% CI)
**Methane Super-emitters**[c]	0.97 (0.91, 1.01)	0.99 (0.94, 1.04)	1.32 (1.14, 1.54)	1.06 (0.97, 1.16)	0.99 (0.95, 1.03)
**Active Oil & Gas Wells**[d]	0.99 (0.94, 1.04)	1.09 (1.03, 1.16)	1.43 (1.21, 1.70)	1.11 (1.00, 1.24)	1.02 (0.97, 1.07)

a. From mixed negative binomial models for frequency of triptans, neurology visits, and migraine-specific urgent care visits. All models included a random intercept for county, adjusted for individual-level age category (18-29, 30-44, 45-54, 55-64, ≥ 65), race/ethnicity (Hispanic, non-Hispanic Asian, non-Hispanic-Black, non-Hispanic White, and non-Hispanic other), sex, Medicaid use, number of primary care visits per person-year during the study period, and block group-level population density and poverty.

b. From mixed logistic models for ≥ 1 ED migraine visit during the study period and MPA score > 100. All models included a random intercept for county, adjusted for individual-level age category (18-29, 30-44, 45-54, 55-64, ≥ 65), race/ethnicity (Hispanic, non-Hispanic Asian, non-Hispanic-Black, non-Hispanic White, and non-Hispanic other), sex, Medicaid use, number of primary care visits per person-year during the study period, and block group-level population density and poverty.

c. Coefficient compares any methane super-emitter within 10km versus none.

d. Coefficient compares any active oil & gas wells within 10km versus none.
